# Peer Review of “In-hospital Mortality and the Predictive Ability of the Modified Early Warning Score in Ghana: Single-Center, Retrospective Study”

**DOI:** 10.2196/30787

**Published:** 2021-07-12

**Authors:** Lincoln Sheets

**Affiliations:** 1 School of Medicine University of Missouri Columbia, MO United States

**Keywords:** modified early warning score, MEWS, AVPU scale, Korle-Bu Teaching Hospital, KBTH, Ghana, critical care, vital signs, global health

*This is a peer-review report submitted for the paper “In-hospital Mortality and the Predictive Ability of the Modified Early Warning Score in Ghana: Single-Center, Retrospective Study”*

## Round 1

### General Comments

This paper [1] describes a study of the modified early warning score (MEWS) and the limited MEWS (LMEWS) instruments for predicting mortality in a tertiary hospital in Ghana.

### Specific Comments

#### Major Comments

1. The two objectives were not described precisely nor were they carefully tied to the methodology. For example, the first objective refers to both “prediction” and “detection” of “deterioration.” It is not clear whether the methodology measures prediction or detection, and it is not clear how deterioration is defined. Mortality is prominent in the results, so this paper might be using mortality as a synonym of deterioration, but that is not clear. In addition, both objectives refer to MEWS, but the results give equal attention to MEWS and LMEWS; it is not clear whether LMEWS is a synonym for the “physiologic measures currently monitored” in the second objective statement; otherwise, LMEWS should be added to both objective statements along with MEWS. In either case, “physiologic measures currently monitored” should be carefully and clearly defined before being used in a statement of objectives.
2. Several statistical measures and tests were reported without being described or explained. I am familiar with some of them, such as the C-statistic, but a reader who is not would need some context for the numbers 0.838 and 0.833—something along the lines of, “where 1.000 means perfect accuracy and 0.500 means perfectly random associations (or ‘the flip of a coin’).” I am not able to suggest explanations for the Pearson chi-square value or the Hosmer-Lemeshow goodness-of-fit test, or the *P* value of the Hosmer-Lemeshow goodness-of-fit test because I am not familiar with this particular measure. Unfortunately, the reporting of the results did not explain the measure at all.
3. The order of MEWS and LMEWS results is completely inconsistent; please always report LMEWS before MEWS or always report MEWS before LMEWS.

#### Minor Comments

1. The grammar and punctuation should be edited throughout; for example, the second sentence of the *Abstract* contains an extraneous semicolon, and the third sentence of the *Abstract* contains an extraneous comma.
